# Optimising workforce competence in home care services: a scoping review protocol

**DOI:** 10.1136/bmjopen-2026-118351

**Published:** 2026-08-27

**Authors:** Marianne Mareliussen, Kari Ingstad, Mojtaba Vaismoradi, Sinikka Lotvonen, Trude Anita Hartviksen

**Affiliations:** 1 Vestvågøy Municipality, Leknes, Norway; 2 Faculty of Nursing and Health Sciences, Nord University, Bodø, Norway; 3 Faculty of Nursing and Health Sciences, Nord University, Campus Levanger, Levanger, Norway; 4 Faculty of Nursing and Health Sciences, Nord University - Bodø, Bodø, Nordland, Norway; 5 Faculty of Science and Health, Charles Sturt University, Albury, New South Wales, Australia; 6 GeroNursing Centre, Research Unit of Health Sciences and Technology, University of Oulu, Oulu, Finland; 7 Center for Care Sciences, North, UiT, The Arctic University of Norway, Tromsø, Troms, Norway

**Keywords:** Health Workforce, Nursing research, Health Services, Human resource management

## Abstract

**Abstract:**

**Introduction:**

Home care services face increasing challenges driven by population ageing, ageing-in-place policies, growing complexity of care needs among home care recipients and shortages of qualified personnel. These developments underscore the need to better use the available workforce and its competencies. Workforce composition refers to the skill mix and organisational structures of home care services, including professional backgrounds, team configurations and work scheduling practices. Competence utilisation refers to how effectively health personnel apply their skills and qualifications in service delivery. This scoping review aims to map existing knowledge on how workforce composition and professional skill sets influence (1) the organisation of tasks, roles and responsibilities and (2) competence utilisation in home care services. By examining the relationship between workforce organisation and competence use, the review seeks to identify factors that support or constrain the optimal use of workforce competence in home care services.

**Methods and analysis:**

This scoping review will be conducted according to the Arksey and O’Malley methodological framework as refined by Levac. The review process will be guided by the Joanna Briggs Institute methodology for scoping reviews. The review has been conducted between April and August 2026. The literature search will be conducted on MEDLINE, EBSCOhost CINAHL, Scopus, ProQuest and Embase to include peer-reviewed primary research studies published in scientific journals. The search will also be supplemented by Google Scholar to identify additional and grey literature in order to improve search coverage. The search strategy has been developed with support from an experienced university librarian. Using the Population–Concept–Context (PCC) framework, inclusion criteria are: (1) employees in home care services, (2) workforce organisation and competence utilisation and (3) municipal or district-based home care services. Exclusion criteria are: (1) volunteers or staff without care responsibilities, (2) studies focusing only on patient outcomes without addressing competence or workforce structure and (3) institutional or hospital-based settings. An online platform will be used to share search results and perform record screenings by two reviewers independently to enhance accuracy and reliability. Data from included sources will be charted and synthesised narratively, supported by the Preferred Reporting Items for Systematic Reviews and Meta-Analysis extension for Scoping Reviews flow diagram (PRISMA-ScR).

**Ethics and dissemination:**

The scoping review will not require ethics approval, as all data will be obtained from publicly available literature. The review authors will adhere to principles of honesty and transparency throughout the data extraction and reporting processes and enhance the trustworthiness of the synthesised findings. Findings will be submitted for publication in a peer-reviewed journal and disseminated through presentations at relevant conferences.

**STRENGTHS AND LIMITATIONS OF THIS STUDY**

- A multidisciplinary research team, including stakeholders and decision-makers, will be actively involved throughout the review process.
- A rigorous scoping review methodology and a comprehensive search strategy, developed in collaboration with a university librarian, will be applied.
- Knowledge gaps will be identified to inform future strategies for optimising competence utilisation in home care services amid increasing care demands.
- The review adopts a broad workforce perspective by including the entire home care services workforce, not only nurses. This enables an exploration of how workforce composition influences roles, task distribution and competence utilisation, which is particularly important in the context of ongoing and anticipated shortages of qualified nursing personnel.
- A potential limitation is the broad scope of workforce composition, which includes diverse competencies and personnel groups, making consistent definitions and precise categorisation across studies challenging.

## Introduction

 Home care services include a wide range of health and social services, encompassing nursing care, care aids, rehabilitation and preventive home visits, that support older adults and individuals with chronic health conditions to live at home.^1^ Demographic changes including population ageing, together with ageing-in-place policies and healthcare reforms promoting community-based care, have increased the reliance on services delivered in home and community settings.^2–4^ International evidence points to a rising demand for home and long-term care, as the WHO projects that the global population aged 60 and over will reach 2.1 billion by 2050, and the Organisation for Economic Co-operation and Development (OECD) reports that more than one-third of adults across 29 OECD countries live with a long-standing illness or health problem.^5 6^ The proportion of people aged 80 years and older is expected to more than triple during the same period, further intensifying demand for complex care in community and home settings and the need to optimise workforce capacity and competence utilisation.^7^ As a result, home care services are increasingly required to meet complex and diverse care needs.^8–10^ This development places growing demands on healthcare personnel, who require extensive and up-to-date knowledge across a wide range of conditions, together with the competence needed to perform complex clinical tasks and adapt care to changing patient situations.^11^ Globally, the health workforce was estimated at over 70 million in 2020, but a shortage of approximately 11 million health workers is projected by 2030.^12 13^ Across OECD countries, the share of long-term care workers in total employment rose only modestly from 1.7% in 2011 to 1.9% in 2021, indicating that workforce growth has not kept pace with rising care needs.^14^ In the context of an anticipated shortage of health personnel and rising demand for services, optimal use of competence will be crucial.^15^

At the organisational level, ensuring high-quality care requires structures that balance standardisation with flexibility and professional discretion.^16 17^ In this context, standardisation refers to organisational procedures, guidelines, routines and work processes designed to promote consistency, quality and patient safety.^18^ At the same time, home care services require flexibility and professional judgement, as care is delivered across diverse home environments and must be adapted to varying, complex and often changing patient needs.^19^ Since no universal organisational and staffing model exists, workforce structures and skill-mix should be adapted to local needs.^20^ In such settings, where healthcare providers frequently work independently but depend on effective coordination, interdisciplinary collaboration can be central to ensuring continuity and comprehensiveness of care.^21^ Interdisciplinary collaboration can strengthen providers’ ability to meet patient needs,^22^ emphasising the value of organisational systems that support coordinated and team-based care. However, limited knowledge exists regarding how organisational structures can facilitate such collaboration and support effective competence utilisation.^23^ Addressing this gap constitutes a key focus of the present review.

The home care services workforce comprises a wide range of personnel with varying levels of training, professional regulation and responsibilities. Within this workforce, nurses constitute one of the largest groups of regulated healthcare professionals involved in home care services.^8 24^ The literature applies a broad and sometimes overlapping set of terms to describe these personnel, including community health workers, healthcare assistants, licensed practical nurses and lay health workers.^8 25^ These categories are not always consistently defined, and distinctions between professional, licensed, regulated and unregulated roles may vary across contexts and healthcare systems. In this review, workforce composition is defined as the configuration of personnel within home care services who contribute to the delivery of daily care. This includes the skill mix, understood as the distribution of professional backgrounds and roles within the workforce, as well as organisational structures such as team configurations and work scheduling practices. Together, these elements capture both the professional diversity of the workforce and the structural organisation of care delivery.^20 26^ Workforce composition influences how tasks, roles and responsibilities are distributed across personnel groups. In response to workforce shortages and increasing service demands, healthcare systems have adopted strategies such as task shifting, role expansion and upskilling of healthcare assistants.^27–29^ While task shifting can improve service capacity, it raises questions about training, workload and role boundaries.^30 31^ Effective implementation therefore depends not only on the competencies of individual workers but also on how teams are organised and supported. Studies suggest that deliberate team composition and collaboration between nurses and healthcare assistants can contribute to improved quality of care and job satisfaction, whereas unclear role definitions and insufficient organisational support may hinder effective care delivery.^20 24 28 32^ In this context, competence utilisation refers to the extent to which health personnel are able to apply their skills and qualifications in clinical practice to support service delivery. This understanding is informed by literature on scope of practice, skill mix and task allocation, which emphasises how workforce composition and the distribution of roles influence the use of available competencies.^22 33^ This review aims to map the relationship between workforce composition and competence utilisation by examining how different workforce configurations are associated with the distribution of tasks, roles and responsibilities in home care services.

Varying organisational contexts, with different levels of care complexity, require broader and more specialised competencies among nursing staff.^34 35^ These competencies may include advanced clinical assessment, chronic disease management, care coordination, clinical decision-making, interprofessional collaboration and the ability to respond to rapidly changing patient conditions. In home care services, where care is delivered in dynamic and often unpredictable environments, optimising competence utilisation is increasingly important, as it requires interdisciplinary coordination and competencies aligned with complex population health needs.^36^ This complexity is multifaceted and driven by increasing reliance on home care services, a growing proportion of individuals with chronic conditions and persistent workforce shortages, which together place increasing demands on care providers and service delivery.^6 37^ The nurses’ roles have expanded to include advanced assessments, coordination and negotiation of responsibilities.^38 39^ However, organisational structures, such as standardised service allocation systems, predefined task schedules and administrative routines, may limit professional discretion by reducing healthcare professionals’ opportunities to adapt care to individual patient needs.^40 41^ Similarly, external control mechanisms, including regulatory requirements, performance targets and managerial oversight, may restrict adaptive capacity, which refers to the ability of healthcare professionals and organisations to adjust care processes in response to changing circumstances and patient needs. Adaptive capacity is considered a key component of resilient healthcare services.^42 43^

Research on staffing, competence utilisation and task distribution has primarily been conducted in hospital settings.^32^ Therefore, evidence from home care services remains limited and fragmented, particularly with regard to the roles of healthcare assistants and the evolving responsibilities of home care nursing staff.^35 38 44 45^ While a recent integrative review by Ranta and Kaunonen^8^ examined competence requirements for nurses in home care, less is known about how workforce composition and organisational structures influence the utilisation of competence across the broader home care workforce. Given the complexity of home care services, a more comprehensive understanding is needed of how organisational structures, workforce composition and task distribution influence competence utilisation across different contexts.^8^ This scoping review adopts a broader workforce perspective by including multiple personnel groups and examining the relationship between workforce composition, task distribution, roles and responsibilities and competence utilisation in home care services. This scoping review generates valuable insights into the organisation of competence in home care services, which can inform policies and theoretical frameworks related to the distribution of tasks, roles and responsibilities in the home care context.

## Methods and analysis

The scoping review will follow the six-stage framework of Arksey^46^ enhanced by Levac *et al*.^47^

### Stage 1: identifying the research question

The review question has been formulated based on the Population–Concept–Context (PCC) framework in line with the Joanna Briggs Institute (JBI) approach^48^ (table 1) as follows:

What does the international literature suggest about organisation and composition of workforce in home care services, examining how these elements shape the distribution of tasks and responsibilities and competence utilisation?

**Table 1:** PCC framework, key concepts and keywords in this review

Framework	Population (P)	Concept (C)	Context (C)
Definition	Workforce refers to staff employed in home care services who provide direct care to users. They are a broader range of formal care personnel such as RNs, LPNs/enrolled nurses, home care assistants, care aides, support workers and allied health professionals involved in home-based care delivery, when they contribute to direct service provision.	Workforce organisation, workforce composition, distribution of tasks, roles and responsibilities, utilisation of competence.	Municipal or district-based home care services that deliver healthcare to individuals in their own homes to support health, functional ability and independence. These services include preventive, rehabilitative, nursing and personal care activities, as well as assistance with activities of daily living.
Inclusion criteria	Registered nurses, healthcare assistants, licensed practical nurses and other licensed and unlicensed staff involved in home care services.	Studies discussing healthcare workforce composition, skill mix or role optimisation.	Studies focused on home-based healthcare or municipal/community care.
Exclusion criteria	Volunteers and employees without care responsibilities.	Studies on patient outcomes without reference to workforce structure or competence.	Studies exclusively on institutional or hospital-based care, hospital-at-home services, transitional care services.
Keywords	Healthcare Personnel, Nurse*, Healthcare Support Worker*, Assistive Personnel*, Healthcare Assistant*	Allocation of Resources, Organizational Efficiency. Professional Delegation, Health Care Team*	Home-based Healthcare, Community Health Services
MeSH	Health Personnel Nurse Practitioners Allied Health Personnel Nursing Staff	Health Workforce, Clinical Competence, ‘Scope of practice’, Efficiency, Organizational, Resource Allocation, ‘Personnel Staffing and Scheduling’, Task Shifting, Professional Role, Delegation, Professional Nursing, Team, Patient Care Team	Home Care Services, Home Health Nursing

LPNs, licensed practical nurses; MeSH, Medical Subject Headings; PCC, Population–Concept–Context; RNs, registered nurses.

### Stage 2: identifying relevant literature

The search strategy will be developed in collaboration with the review team and supported by an experienced university librarian to ensure methodological rigour and the use of appropriate keywords for ‘Home Care Services’, ‘Health Personnel’, ‘Workforce’ and ‘Professional Competence*’* (online supplemental file 1). Electronic searches will be conducted on the following databases: MEDLINE, EBSCOhost CINAHL, Scopus, ProQuest and Embase in English language. Database-specific strategies will be adapted to each platform, applying the Boolean method to combine text words related to the review keywords and concepts. To capture additional sources, grey literature consisting of guidelines, governmental reports, policy documents and professional recommendations, primarily through targeted Google searches and websites of health authorities relevant to the review topic in English and Scandinavian languages will be searched. In addition, backward and forward citation searching^49^ will be conducted to improve search coverage. The reference lists of all included studies and relevant review articles will be screened to identify potentially relevant sources. Studies published from 2015 onwards will be included to capture contemporary literature reflecting current developments in home care services, including workforce composition, competence utilisation, skill-mix strategies, task allocation and service organisation. This timeframe was selected to ensure that the review reflects current care practices, organisational structures and workforce challenges within home care services.^13 35^ Furthermore, this study employs a scoping review methodology aimed at mapping key concepts, evidence types and knowledge gaps, rather than synthesising findings in the manner of a traditional systematic review.

### Stage 3: study selection

All identified records will be imported into the online platform of Rayyan, for the collaborative screening and selection of studies, where duplicates will be removed prior to screening. Study selection will follow a two-stage screening process: (1) Title and abstract screening, conducted independently by four reviewers, using the predefined inclusion and exclusion criteria based on the PCC framework. (2) Full-text screening of potentially relevant studies to assess eligibility.

Any disagreements between reviewers will be resolved through discussion or consultation with a third reviewer when necessary. Reasons for exclusion at the full-text stage will be documented. The selection process will be reported using the Preferred Reporting Items for Systematic Reviews and Meta-Analysis extension for Scoping Reviews (PRISMA-ScR) flow diagram.

### Stage 4: charting the data

A standardised data-charting form will be developed and pilot-tested on a sample of studies to ensure consistency and relevance. The form will be refined iteratively as needed. Data will be charted independently by two reviewers. The data extraction form will include, but not be limited to: bibliographic details (author, year, country), study purpose and design (table 2), characteristics of the workforce (eg, profession, composition, skill mix), organisational structures relevant to task distribution, descriptions of tasks, roles and responsibilities, reported impacts on competence utilisation, key findings relevant to the research question (table 3). Data charting will focus on capturing both descriptive characteristics and thematic elements related to workforce organisation and competence utilisation.

**Table 2:** Proposed data charting form of the general characteristics of included studies

Authors, year, country	Participants number and setting	Aim	Study design	Study measurements	Main results/finding	Authors conclusion
-	-	-	-	-	-	-

**Table 3:** Proposed data charting form of workforce characteristics, organisational structures and competence utilisation

Study	Workforce characteristics	Organisational structure	Competence utilisation	Conclusion
	Profession, composition, skill mix	Task distribution, descriptions of tasks, roles and responsibilities	Use of professional competence in practice	

### Stage 5: collating, summarising and reporting the results

The findings will be synthesised following the JBI approach to scoping reviews, which includes descriptive and thematic analysis. The analysis will involve descriptive mapping of study characteristics, workforce types and organisational models, thematic synthesis to identify patterns related to task distribution, competence utilisation and workforce composition, highlighting gaps in the existing evidence base.

The results will be presented in tables, figures and narrative summaries to provide an accessible overview of how organisational structures and workforce composition influence competence utilisation in home care services. The review will adhere to the PRISMA-ScR reporting guidelines.

### Stage 6: consultation

This stage aims to enhance the methodological rigour of the review by consulting relevant stakeholders to identify additional sources or research that may be missed. Relevant stakeholders include managers and employee representatives from home care services in the collaborating municipalities, as well as academic partners involved in the wider research project. This review forms the first part of a larger research project involving qualitative interviews with stakeholders from three collaborative municipalities and a university in Norway and Finland, which facilitates a broad perspective and a close link to the practice field, thereby enabling the integration of experiential and contextual knowledge. In this review, relevant stakeholders will be invited to participate in digital meetings to present and discuss preliminary findings, with the aim of validating the findings and help identify priorities for the subsequent qualitative study. The international scope of the review is intended to support the next phase of the project by providing knowledge on how workforce composition, competence utilisation and task distribution are addressed across different health and care systems. Including international qualitative studies enables the identification of both transferable lessons and context-specific factors that may influence workforce organisation and service delivery in home care. Furthermore, the inclusion of international qualitative studies in this review captures the perspectives, experiences and priorities of relevant stakeholders, such as patients, informal caregivers and healthcare professionals, thereby providing contextual and practice-based insights. In this way, the consultation stage will complement the systematic review process and strengthen the relevance and applicability of the study findings.

### Patient and public involvement statement

Patient or public were not involved in this part of the research.

## Ethics and dissemination

The scoping review does not require ethical approval, as it will exclusively use data from publicly available and previously published literature. The review authors will adhere to principles of honesty and transparency throughout the data extraction and reporting processes and enhance the trustworthiness of the synthesised findings. To uphold these principles, the research team will document and report all steps in the review process in a reproductive and transparent manner.^50^ The results of the scoping review will be reported in an article submitted for publication in an international peer-reviewed journal and presented at relevant conferences. In addition, the findings may also inform policy and practice by providing an evidence-based overview of workforce composition and competence utilisation in home care services, thereby supporting decision-making and service development.

## Supplementary material

10.1136/bmjopen-2026-118351 online supplemental file 1
