# Supplementary material for: Optimising workforce competence in home care services: a scoping review protocol
Source: BMJ Open. 2026 Aug 27;16(8):e118351. doi: 10.1136/bmjopen-2026-118351 (PMC13536040; doi:10.1136/bmjopen-2026-118351)
Supplement: Supplementary file 1 [file bmjopen-2026-118351.reviewer_comments.pdf]

# PEER REVIEW HISTORY

BMJ Open publishes all reviews undertaken for accepted manuscripts. Reviewers are asked to complete a checklist review form and are provided with free text boxes to elaborate on their assessment. These free text comments are reproduced below.

## ARTICLE DETAILS

### Title (Provisional)

Optimizing Workforce Competence in Home Care Services: A Scoping Review Protocol

### Authors

Mareliussen, Marianne; Ingstad, Kari; Vaismoradi, Mojtaba; Lotvonen, Sinikka; Hartviksen, Trude Anita

---

## VERSION 1 - REVIEW

---

| | |
|---|---|
| **Reviewer** | **1** |
| **Name** | **Rahja, Miia** |
| **Affiliation** | **Flinders University** |
| **Date** | **14-Apr-2026** |
| **COI** | **None** |

---

Thank you for the opportunity to review this scoping review protocol about "Optimizing Workforce Competence in Home Care Services". The manuscript addresses an important topic, however, is currently underdeveloped. Key concepts lack clarity, the rationale for the review is insufficiently supported, and there are gaps in the description of methods and data charting. I would recommend major revisions to improve conceptual clarity, methodological transparency, and alignment between the title, aims, and planned analyses. Below are key points from my review:

Abstract

Introduction: This section is difficult to follow and does not clearly reflect the focus suggested by the title. The authors state that the review aims to map current knowledge on how the composition and professional skill sets of health personnel in home care services influence competence utilisation and the distribution of tasks, roles, and responsibilities. However, what is what is meant by "composition" (e.g. staffing mix, professional diversity, team structure)? Also, "competence utilisation" is not defined or operationalised, making it difficult to understand how this concept will be examined.

The relationship between workforce composition, competence utilisation, task distribution, and the stated aim of optimising workforce competence is not clearly articulated.

I recommend that the authors break this into clearer, more focused statements and explicitly explain what concepts are being mapped, how they relate to one another and how mapping these relationships contributes to optimising workforce competence in home care services.

Methods

Key methodological details are missing from the abstract, including, the time frame for the review (start and end dates), the types of studies to be included, inclusion and exclusion criteria; and whether the review has commenced or is planned.

Including these details would improve transparency and alignment with scoping review reporting standards.

Strengths and limitations

The strengths listed are generic and not clearly specific to this review. The authors should articulate what this review adds that has not already been addressed in existing literature and why this particular focus (e.g. competence utilisation and task distribution in home care) is novel or important.

No limitations are identified. The authors should include anticipated limitations.

Introduction

Paragraph 1: The first paragraph references growth in the long-term care workforce but does not provide any data on rising care needs. Including statistics on demographic ageing, chronic disease prevalence, or increasing demand for home care would better justify the importance of the topic.

It may also be helpful to briefly describe examples of home care services (e.g. nursing care, personal support, rehabilitation, preventive visits) to clearly situate the review context.

Paragraph 2: The discussion of interdisciplinary collaboration could be further developed. If research suggests that interdisciplinary care strengthens providers' ability to meet patient needs, the authors should clarify why this matters for home care services; and how this informs the review aims. The "so what" of this paragraph is currently unclear.

Paragraph 3: This paragraph would benefit from revision for clarity and logical flow. The first sentence refers to a wide range of personnel and then uses "however," but the contrast is not clear. Additionally, this paragraph would be better grounded if concrete examples of home care services and workforce roles were already introduced earlier in the introduction.

Paragraph 4: The manuscript refers to increasing care complexity but does not provide examples. Nor is it explained why home care providers are increasingly caring for more complex clients. Providing this context would strengthen the argument for examining workforce competence.

Overall, the introduction lacks specific empirical and practice-based examples that clearly demonstrate why examining professional skill sets and competence utilisation in home care is necessary and timely.

Methods and Analysis

Table 1: In the cell describing population/definition, the authors could consider including allied health professionals and other relevant occupational groups, rather than focusing on nursing and care staff.

Stage 2: The justification for the selected time frame seems to be specific to a single national context. The authors should clarify why this rationale is appropriate for an international scoping review. Additionally, the cited references do not clearly support the proposed justification.

The authors should also explicitly state how this review differs from Ranta and Kaunonen (2024), in terms of scope, concepts, or methodological approach.

Stage 4 / Table 2 (Data charting): It is not clear how the planned data charting will capture workforce characteristics (e.g., profession, composition, skill mix), organisational structures related to task distribution, descriptions of tasks, roles, and responsibilities and reported impacts on competence utilisation. These elements are referenced in the text but not adequately reflected in the proposed charting table. Perhaps revise to ensure alignment between the stated aims and the data to be extracted.

Stage 6 (Consultation): The manuscript states that stakeholder consultation will occur, but it is unclear who the relevant stakeholders are and what strategies will be used to engage with them.

Thank you again for the opportunity to review this work. I wish the authors all the best with the research.

---

| | |
|---|---|
| **Reviewer** | **2** |
| **Name** | **Saari, Margaret** |
| **Affiliation** | **Saint Elizabeth Research Centre** |
| **Date** | **23-Apr-2026** |
| **COI** | **None** |

---

This scoping review addresses an important topic related to home care workforce research and practice by mapping what is known in the literature about who is providing care, how that care is organized and whether or not these structures result in optimal utilization of the skills various members of the workforce possess. With revisions to strengthen conceptual clarity, address definitional concerns and better situate the work in the broader literature, the manuscript has strong potential to make a valuable contribution to the topic.

Introduction

The authors provide a strong introduction to home care, why the published literature would benefit from synthesis and current practice and operational challenges. However, key concepts relevant to the research question are not adequately introduced or defined. Critically, the authors need to define what is meant by “optimal use of competence” as this is not a commonly used term and the concept is central to the review. Further,

Methods

While the research question (RQ) addresses an important policy- and practice relevant question as noted above, it would benefit from refinement to improve clarity, precision and alignment with the aims of a scoping review (e.g. to map and describe). As written, it is conceptually dense with multiple dimensions. Ensure all terms included in the RQ are clearly defined in your introduction including "organization", "composition of workforce" and "utilization of competence" as these could refer to many different things. For example, organization could refer to governance, staffing models, care delivery structures or management arrangements. Clarifying the constructs will support enhanced transparency and usability for this scoping review.

PCC Table

Given the broad definition used by the authors to define the home care workforce “we therefore adopt an inclusive approach, encompassing all personnel employed in home care services who contribute to the delivery of daily care”, keywords for the population should be expanded both to adequately capture the breadth of terms used for unregulated personnel as well as the broader interdisciplinary team (e.g., OT, PT, SLP, RD etc). Alternatively, the authors should refine their positioning to specify they are interested in only those providing nursing care (including nurse practitioners/advanced practice nurses, registered nurses, practical nurses, unregulated personnel).

Aligned to comments regarding the RQ being conceptually dense, the Concept criteria reflect that complexity and may require further refinement once the concepts are more clearly delineated. As currently listed, this concept is very broad without sufficient depth in search strategy to effectively map the literature related to these concepts.

For the Context – you will want to consider if hospital-to-home or transitional care programs would be included as they are not exclusively community based nor hospital based and so would not have a clear result based on current inclusion/exclusion criteria. Further, the home care literature is often not well indexed and so a more complex strategy may be required to capture the home care context while simultaneously excluding a vast amount of literature related to “care homes” (e.g. nursing home care).

In its current form, this search is likely to return large numbers of peer reviewed papers, which makes me question the feasibility of further grey literature searches. However, if this is to be an important component of the review, further details regarding the process for

searching as well as identifying relevant health authorities would be appreciated. Will these be international or restricted to the author's primary location?

No comments were provided re: types of sources or language restrictions – please include. Further details re: the relevant changes in municipal home care services (is this location specific?) would help support the timeframe limitations.

How will relevant stakeholders be consulted throughout the scoping review process? Were they involved in developing and defining the research question, if so, how? Given the focus on stakeholder involvement, is the only planned use for the review academic publication and presentation or is the intention for broader policy or practice impact?

In addition to your review of expanding roles of unregulated care providers, consider reviewing the development of Canada's updated home health nursing competencies to inform this work: http://pubmed.ncbi.nlm.nih.gov/40197356/

Editorial

- Check referencing style as some in-text citations were noted as not following formatting requirements)

---

## VERSION 1 - AUTHOR RESPONSE

### Reviewer 1

Dr. Miia Rahja, Flinders University
Comments to the Author:
Thank you for the opportunity to review this scoping review protocol about "Optimizing Workforce Competence in Home Care Services". The manuscript addresses an important topic, however, is currently underdeveloped. Key concepts lack clarity, the rationale for the review is insufficiently supported, and there are gaps in the description of methods and data charting. I would recommend major revisions to improve conceptual clarity, methodological transparency, and alignment between the title, aims, and planned analyses. Below are key points from my review:

1. Abstract

   Introduction: This section is difficult to follow and does not clearly reflect the focus suggested by the title. The authors state that the review aims to map current knowledge on how the composition and professional skill sets of health personnel in home care services influence competence utilisation and the distribution of tasks, roles, and responsibilities. However, what is what is meant by "composition" (e.g. staffing mix, professional diversity, team structure)? Also, "competence utilisation" is not defined or operationalised, making it difficult to understand how this concept will be examined.
   ***Answer****: Thank you for your helpful comment. We have clarified the concepts of composition and competence utilization by adding their definitions in the Introduction, lines 26–30, page 2.*

The relationship between workforce composition, competence utilisation, task distribution, and the stated aim of optimising workforce competence is not clearly articulated.
I recommend that the authors break this into clearer, more focused statements and explicitly explain what concepts are being mapped, how they relate to one another and how mapping these relationships contributes to optimising workforce competence in home care services.
***Answer:*** *Thank you for this comment; we agree that the relationships between workforce composition, competence utilization, task distribution, and their contribution to optimizing workforce competence needed clearer articulation. We have revised the Introduction to address this by providing a more explicit explanation of how these concepts relate to one another and how they will be explored in this review, lines 32–37, page 2.*

2. Methods
   Key methodological details are missing from the abstract, including, the time frame for the review (start and end dates), the types of studies to be included, inclusion and exclusion criteria; and whether the review has commenced or is planned.
   Including these details would improve transparency and alignment with scoping review reporting standards.
   ***Answer:*** *The methods and analysis paragraph have been supplemented with methodological details describing the timeframe, as well as the inclusion and exclusion criteria: Lines 41-42, 43-44 and 46-51, page 2.*

3. Strengths and limitations
   The strengths listed are generic and not clearly specific to this review. The authors should articulate what this review adds that has not already been addressed in existing literature and why this particular focus (e.g. competence utilisation and task distribution in home care) is novel or important.
   No limitations are identified. The authors should include anticipated limitations.
   ***Answer:*** *Thank you for your comment. We agree that the strengths should be more specifically articulated and that anticipated limitations should be included. We have revised the section to clarify how this review contributes to the international literature and its specific focus. In addition, we have now included a description of the anticipated limitations of the review, lines 64–77, page 3.*

4. Introduction

   Paragraph 1: The first paragraph references growth in the long-term care workforce but does not provide any data on rising care needs. Including statistics on demographic ageing, chronic disease prevalence, or increasing demand for home care would better justify the importance of the topic.
   It may also be helpful to briefly describe examples of home care services (e.g. nursing care, personal support, rehabilitation, preventive visits) to clearly situate the review context.

***Answer***: *We have added relevant statistics on demographic ageing, chronic disease prevalence, and the increasing demand for home care to better justify the importance of the topic. In addition, we have included examples of home care services to more clearly situate the context of the review. Lines 80–100, page 3-4.*

5. Paragraph 2: The discussion of interdisciplinary collaboration could be further developed. If research suggests that interdisciplinary care strengthens providers' ability to meet patient needs, the authors should clarify why this matters for home care services; and how this informs the review aims. The "so what" of this paragraph is currently unclear.
***Answer***: *We agree that the relevance of interdisciplinary collaboration and its implications for home care services need to be clarified. We have therefore revised the paragraph to better articulate the relationship between interdisciplinary collaboration and patient care, and how this informs the aim of the review (lines 109–115 and 118–120, page 4)*

6. Paragraph 3: This paragraph would benefit from revision for clarity and logical flow. The first sentence refers to a wide range of personnel and then uses "however," but the contrast is not clear. Additionally, this paragraph would be better grounded if concrete examples of home care services and workforce roles were already introduced earlier in the introduction.
***Answer***: *We have revised the paragraph to strengthen the connections between sentences and improve overall coherence. In addition, we have included concrete examples of home care services and workforce roles earlier in the Introduction to better ground the discussion. Lines 121–153, pages 4–5; lines 80–81, page 3.*

7. Paragraph 4: The manuscript refers to increasing care complexity but does not provide examples. Nor is it explained why home care providers are increasingly caring for more complex clients. Providing this context would strengthen the argument for examining workforce competence.
***Answer***: *We have added examples illustrating why home care providers are caring for more complex clients to strengthen the rationale for examining workforce competence, lines 154–162, page 5.*

8. Overall, the introduction lacks specific empirical and practice-based examples that clearly demonstrate why examining professional skill sets and competence utilisation in home care is necessary and timely.
***Answer***: *Thank you for this comment. We agree that the introduction needed stronger empirical and practice-based examples to better demonstrate the relevance of examining professional skill sets and competence utilization in home care services. We have revised the Introduction to strengthen this aspect and provide clearer justification for the focus of the review.*

9. Methods and Analysis

Table 1: In the cell describing population/definition, the authors could consider including allied health professionals and other relevant occupational groups, rather than focusing on nursing and care staff.
***Answer****: We agree that it is important to include a broader range of relevant occupational groups. We have revised the population definition in Table 1 to include allied health professionals and other relevant groups involved in home care, line 189, page 6.*

Stage 2: The justification for the selected time frame seems to be specific to a single national context. The authors should clarify why this rationale is appropriate for an international scoping review. Additionally, the cited references do not clearly support the proposed justification.
The authors should also explicitly state how this review differs from Ranta and Kaunonen (2024), in terms of scope, concepts, or methodological approach.
***Answer****: We have revised the manuscript to better explain and support the rationale for the chosen time period using relevant literature. In addition, we have clarified how this review differs from Ranta and Kaunonen (2024) in terms of scope, key concepts, and methodological approach. Lines 211–226, page 7-8.*

Stage 4 / Table 2 (Data charting): It is not clear how the planned data charting will capture workforce characteristics (e.g., profession, composition, skill mix), organisational structures related to task distribution, descriptions of tasks, roles, and responsibilities and reported impacts on competence utilisation. These elements are referenced in the text but not adequately reflected in the proposed charting table. Perhaps revise to ensure alignment between the stated aims and the data to be extracted.
***Answer****: We have therefore Tables 2 and 3 to ensure that key elements—such as workforce characteristics, organizational structures, task distribution, and competence utilization—are more clearly captured in the data extraction process, lines 247–250, page 8-9.*

Stage 6 (Consultation): The manuscript states that stakeholder consultation will occur, but it is unclear who the relevant stakeholders are and what strategies will be used to engage with them.
***Answer****: We agree that greater clarity was needed regarding the stakeholder consultation process. We have therefore revised this section to more clearly describe the relevant stakeholders and the strategies for engaging with them, lines 263–272, page 9.*

Thank you again for the opportunity to review this work. I wish the authors all the best with the research.

**Reviewer 2**

Dr. Margaret Saari, Saint Elizabeth Research Centre

Comments to the Author:

This scoping review addresses an important topic related to home care workforce research and practice by mapping what is known in the literature about who is providing

care, how that care is organized and whether or not these structures result in optimal utilization of the skills various members of the workforce possess. With revisions to strengthen conceptual clarity, address definitional concerns and better situate the work in the broader literature, the manuscript has strong potential to make a valuable contribution to the topic.

1. Introduction
   The authors provide a strong introduction to home care, why the published literature would benefit from synthesis and current practice and operational challenges. However, key concepts relevant to the research question are not adequately introduced or defined. Critically, the authors need to define what is meant by "optimal use of competence" as this is not a commonly used term and the concept is central to the review. Further,
   ***Answer:*** *Thank you for this important observation. We agree that key concepts required clearer definition, and we have therefore revised the Introduction to better define and clarify relevant concepts, including composition and competence utilization, lines 129–135, page 4; lines 145-153, page 5.*

2. Methods
   While the research question (RQ) addresses an important policy- and practice relevant question as noted above, it would benefit from refinement to improve clarity, precision and alignment with the aims of a scoping review (e.g. to map and describe). As written, it is conceptually dense with multiple dimensions. Ensure all terms included in the RQ are clearly defined in your introduction including "organization", "composition of workforce" and "utilization of competence" as these could refer to many different things. For example, organization could refer to governance, staffing models, care delivery structures or management arrangements. Clarifying the constructs will support enhanced transparency and usability for this scoping review.
   ***Answer****: Thank you for this constructive comment. We agree that the research question required greater clarity and alignment with the aims of a scoping review, and that key concepts needed to be more clearly defined. We have therefore revised the Introduction to provide clearer definitions of central concepts, to ensure that the research question in the Methods section is aligned with these definitions.*

3. PCC Table
   Given the broad definition used by the authors to define the home care workforce "we therefore adopt an inclusive approach, encompassing all personnel employed in home care services who contribute to the delivery of daily care", keywords for the population should be expanded both to adequately capture the breadth of terms used for unregulated personnel as well as the broader interdisciplinary team (e.g., OT, PT, SLP, RD etc). Alternatively, the authors should refine their positioning to specify they are interested in only those providing nursing care (including nurse practitioners/advanced practice nurses, registered nurses, practical nurses, unregulated personnel).

***Answer****: We have specified and expanded examples of relevant occupational grou), and incorporated these into Table 1, line 189, page 6.*

4. Aligned to comments regarding the RQ being conceptually dense, the Concept criteria reflect that complexity and may require further refinement once the concepts are more clearly delineated. As currently listed, this concept is very broad without sufficient depth in search strategy to effectively map the literature related to these concepts.
   ***Answer****: We agree that the conceptual scope was broad and required clearer delineation. We have therefore refined the concepts a to improve clarity and alignment, following the definitions provided in the Introduction, thereby strengthening the coherence between the research question, concepts, and search strategy. Lines 80-178, page 3-6*

5. For the Context – you will want to consider if hospital-to-home or transitional care programs would be included as they are not exclusively community based nor hospital based and so would not have a clear result based on current inclusion/exclusion criteria. Further, the home care literature is often not well indexed and so a more complex strategy may be required to capture the home care context while simultaneously excluding a vast amount of literature related to "care homes" (e.g. nursing home care).
   ***Answer****: We have clarified the definition of home care in both the Introduction and Table 1 to more explicitly specify the types of contexts and services included and excluded in this review, line 189, page 6.*

6. In its current form, this search is likely to return large numbers of peer reviewed papers, which makes me question the feasibility of further grey literature searches. However, if this is to be an important component of the review, further details regarding the process for searching as well as identifying relevant health authorities would be appreciated. Will these be international or restricted to the author's primary location?
   ***Answer****: We have refined the scope by clearly focusing on home care services and excluding other care settings, which improves the feasibility of the review. In addition, we have clarified that the grey literature search will include both international sources (English language) and national sources (Scandinavian languages), and have provided further detail on this process in the manuscript. A draft search strategy has also been attached to enhance transparency and reproducibility. Lines 199-204, page 7.*

7. No comments were provided re: types of sources or language restrictions – please include. Further details re: the relevant changes in municipal home care services (is this location specific?) would help support the timeframe limitations.
   ***Answer****: We have included details regarding the types of sources and language restrictions in the manuscript. In addition, we have provided further clarification and supporting information regarding the selected timeframe, including relevant*

*contextual changes in municipal home care services. Line 196-202, page 7; lines 211-218, page 7-8.*

8. How will relevant stakeholders be consulted throughout the scoping review process? Were they involved in developing and defining the research question, if so, how? Given the focus on stakeholder involvement, is the only planned use for the review academic publication and presentation or is the intention for broader policy or practice impact?
   ***Answer**: We have elaborated the strategy for stakeholder involvement, including how and when stakeholders will be consulted throughout the scoping review process. We have also clarified the intended use of the review, highlighting its relevance not only for academic purposes but also for informing policy and practice. Lines 263-272, page 9; lines 287-290, page 10.*

9. In addition to your review of expanding roles of unregulated care providers, consider reviewing the development of Canada's updated home health nursing competencies to inform this work: http://pubmed.ncbi.nlm.nih.gov/40197356/
   ***Answer**: We have considered the referenced work and have included it as a reference. This has helped strengthen the Introduction chapter and further inform and refine the description of the population included in our review. Lines 156-159, page 5.*

---

## VERSION 2 - REVIEW

---

| | |
|---|---|
| **Reviewer** | **1** |
| **Name** | **Rahja, Miia** |
| **Affiliation** | **Flinders University** |
| **Date** | **07-Jul-2026** |
| **COI** | |

---

Thank you for the opportunity to review this revised manuscript. Overall, this version has improved compared with the original submission. However, I would encourage the authors to pay attention to the clarity and readability of the manuscript. Throughout the paper, several concepts and terms are still introduced without sufficient explanation, and several sentences are unnecessarily complex, making the text difficult to follow.

I would also continue to encourage the authors to provide a more detailed description of relevant prior literature, to help readers understand why the current review is needed and what unique contribution it seeks to make.

Abstract - Introduction

I would consider reframing this section and making it more concise. For example: Home care services face increasing challenges driven by population ageing, ageing-in-place policies,

growing complexity of care needs among home care recipients, and shortages of qualified personnel.

Abstract - Methods

The abstract states that the review will be conducted between April and June 2026. Is this correct? If so, it may be too late to publish this protocol.

Introduction

Paragraph 1

I believe this section is referring to ageing-in-place policies, although this is not clear. It may be helpful to state this more explicitly.

I am also uncertain about the statement that increasing complexity of patient care needs leads to transitions from long-term care homes to the community; I would have expected the opposite. Clarification or supporting evidence would be helpful here.

In the sentence beginning "As a result, more people receive home care services...", does this refer to current service use or future demand. If this is a projection, it may be more appropriate to state that more people are expected to need home care services. Similarly, I am unsure how the authors have established that services are becoming more advanced. Would it be better to state that services are increasingly required to meet more complex and diverse care needs?

Paragraph 2

I found the beginning of this paragraph difficult to interpret. The authors should \clarify what is meant by "standardization" in the context of home care services and explain why a balance between standardization, flexibility, and professional discretion is required to support high-quality care?

Paragraph 3

A % for the statement that nurses represent the largest group of health professionals delivering home care services would be helpful.

More broadly, I found this paragraph difficult to follow because it introduces several concepts without clearly explaining how they relate to one another. A few examples are:

- The terminology shifts between "personnel" and "care providers."
- In the sentence "Taken together, the concept captures...", does this refer to the concept of workforce composition?
- In the sentence "Changes in the workforce are closely linked to task shifting as a strategy to address workforce shortages”. Does this refer to changes in the activities that the personnel do as opposed what their training or qualification is?

• The statement "Deliberate team composition and collaboration between nurses and assistants have been shown to be associated with improved quality and job satisfaction" seems disconnected from the preceding discussion about task shifting. Please clarify the relationship. Similarly the relationship to unclear roles and insufficient organisational support needs linking.

• The progression towards the review aim would benefit from a clearer and more logically structured argument.

Paragraph 5

The statement that organisational contexts with greater care complexity require broader and more specialised competencies would benefit from examples. What specific competencies are being referred to?

Similarly, what is meant by "organisational structures can limit professional discretion" and "external control mechanisms restrict adaptive capacity"? These concepts are not clear to readers and would benefit from further explanation.

Paragraph 6

Please check that the reference style is consistent with journal requirements.

I would also have expected to see a more detailed description of the relevant 2024 review in this section rather than in the Methods.

Methods and Analysis

Step 1

The review question could be refined. The phrase: "examining how these elements shape the distribution of tasks and responsibilities, and competence utilization" reads more like an objective than part of the review question. Would it be better to ask: "What does the international literature suggest about the organisation and composition of the home care workforce, and how do these factors influence the distribution of tasks, responsibilities, and competence utilisation?"

Step 2

I remain unconvinced by the rationale provided for the timeframe restriction. The justification appears to reflect a particular national context, which I was unable to identify in the cited reference. It may be sufficient to state that studies from 2015 onwards were included to capture contemporary literature on workforce competency, quality, and efficiency, which appears more consistent with the cited WHO publication.

I would also encourage the authors to make the rationale for the review easier for readers to follow. For example, rather than stating: "While Ranta and Kaunonen [9] focus specifically..."consider briefly describing the review and its key focus before explaining how

the current review differs and addresses an important gap. I would also move this to the end of your introduction section.

Step 6

I suggest identifying the relevant stakeholders earlier in the section. It would also be helpful to specify the country context in which the review findings are intended to be applied and how stakeholders are expected to use the findings.

The rationale for including international qualitative studies could also be developed. While I understand that qualitative studies can provide perspectives, experiences, and priorities from stakeholders, it is not clear why an international perspective is important for this review. For example, are the authors seeking to identify transferable lessons from other countries, compare workforce approaches across health and care systems, or understand the role of contextual factors in workforce organisation and care delivery? Clarifying the value of the international scope would strengthen the rationale for the review.

Thank you for the opportunity to review this revised manuscript. Overall, while this revision has improve the quality of the work, I would specifically suggest the authors focus on the quality of their writing and making reading of their manuscript easy for interested audiences.

---

| | |
|---|---|
| **Reviewer** | **2** |
| **Name** | **Saari, Margaret** |
| **Affiliation** | **Saint Elizabeth Research Centre** |
| **Date** | **29-Jun-2026** |
| **COI** | |

---

Author has addressed my concerns. Looking forward to reading the final article.

---

## VERSION 2 - AUTHOR RESPONSE

1. Reviewer: 2
   Dr. Margaret Saari, Saint Elizabeth Research Centre

   Comments to the Author:
   Author has addressed my concerns. Looking forward to reading the final article.

   *Answer: Thank you for your positive feedback and for your valuable comments during the review process. We greatly appreciate your thoughtful input, which has helped strengthen the manuscript.*

2. Reviewer: 1
   Dr. Miia Rahja, Flinders University

   Comments to the Author:
   Thank you for the opportunity to review this revised manuscript. Overall, this version has improved compared with the original submission. However, I would encourage the authors to pay attention to the clarity and readability of the manuscript. Throughout the paper, several concepts and terms are still introduced without sufficient explanation, and several sentences are unnecessarily complex, making the text difficult to follow.
   I would also continue to encourage the authors to provide a more detailed description of relevant prior literature, to help readers understand why the current review is needed and what unique contribution it seeks to make.

   *Answer: Thank you for your thoughtful and constructive feedback. We appreciate your recognition of the improvements made in the revised manuscript. In response to your comments, we have carefully reviewed the manuscript and made further revisions to improve clarity, readability, and overall flow. We have also expanded and clarified the discussion of relevant prior literature to better articulate the rationale for the review, identify existing knowledge gaps, and highlight the unique contribution of the present study. We believe these revisions have strengthened the manuscript and improved its accessibility for readers. Page 5, line 159-166, line 170-173, line 176-179.*

3. Abstract - Introduction
   I would consider reframing this section and making it more concise. For example: Home care services face increasing challenges driven by population ageing, ageing-in-place policies, growing complexity of care needs among home care recipients, and shortages of qualified personnel.

   *Answer: Thank you for this valuable suggestion. We agree that the introductory section of the abstract could be made more concise and focused. The paragraph has therefore been revised to more clearly emphasize the key drivers affecting home care services, including population ageing, ageing-in-place policies, increasing care complexity, and workforce shortages. The revised text also streamlines the description of the review aim while retaining the key concepts of workforce composition and competence utilization. The manuscript has been updated accordingly. Page 2, lines 23-24, 26-29, 32-34*

4. Abstract - Methods
   The abstract states that the review will be conducted between April and June 2026. Is this correct? If so, it may be too late to publish this protocol.

   *Answer: Thank you for highlighting this issue. You are correct that the dates stated in the abstract needed updating. The time frame has now been revised and corrected in the updated version of the manuscript to accurately reflect the review timeline. We appreciate the reviewer bringing this error to our attention. Page 2, line 39.*

5. Introduction
   Paragraph 1
   I believe this section is referring to ageing-in-place policies, although this is not clear. It may be helpful to state this more explicitly.
   I am also uncertain about the statement that increasing complexity of patient care needs leads to transitions from long-term care homes to the community; I would have expected the opposite. Clarification or supporting evidence would be helpful here.
   In the sentence beginning "As a result, more people receive home care services...", does this refer to current service use or future demand. If this is a projection, it may be more appropriate to state that more people are expected to need home care services. Similarly, I am unsure how the authors have established that services are becoming more advanced. Would it be better to state that services are increasingly required to meet more complex and diverse care needs?

   *Answer: Thank you for this insightful comment. We agree that the original wording could be interpreted as unclear. The text has been revised to explicitly refer to ageing-in-place policies and the broader shift towards community-based care. We have also clarified that increasing complexity of care needs does not itself drive the transition from institutional care to home care services; rather, demographic changes, policy reforms, and efforts to support people living at home have contributed to a greater reliance on community-based services. Furthermore, we have revised the wording regarding service demand and complexity to avoid overstating current trends and to better reflect that home care services are increasingly required to meet more complex and diverse care needs. The relevant section has been updated accordingly. Page 3, lines 80-82, 89-94.*

6. Paragraph 2
   I found the beginning of this paragraph difficult to interpret. The authors should

\clarify what is meant by "standardization" in the context of home care services and explain why a balance between standardization, flexibility, and professional discretion is required to support high-quality care?

*Answer: Thank you for this helpful comment. We agree that the concept of standardization was insufficiently explained in the original manuscript. The paragraph has been revised to clarify that standardization refers to organizational procedures, guidelines, routines, and work processes intended to promote consistency, quality, and patient safety in care delivery. We have also elaborated on why a balance between standardization, flexibility, and professional discretion is necessary in home care services, where healthcare personnel must continuously adapt care to diverse, complex, and changing patient needs across different home environments. The relevant section has been revised accordingly. Page 4. lines 101-106, 112-114.*

7. Paragraph 3
A % for the statement that nurses represent the largest group of health professionals delivering home care services would be helpful.
More broadly, I found this paragraph difficult to follow because it introduces several concepts without clearly explaining how they relate to one another. A few examples are:
• The terminology shifts between "personnel" and "care providers."
• In the sentence "Taken together, the concept captures…", does this refer to the concept of workforce composition?
• In the sentence "Changes in the workforce are closely linked to task shifting as a strategy to address workforce shortages”. Does this refer to changes in the activities that the personnel do as opposed what their training or qualification is?
• The statement "Deliberate team composition and collaboration between nurses and assistants have been shown to be associated with improved quality and job satisfaction" seems disconnected from the preceding discussion about task shifting. Please clarify the relationship. Similarly the relationship to unclear roles and insufficient organisational support needs linking.
• The progression towards the review aim would benefit from a clearer and more logically structured argument.

*Answer: Thank you for this thoughtful and detailed comment. We agree that the original paragraph introduced several interconnected concepts without sufficiently explaining the relationships between them. The paragraph has therefore been substantially revised to improve clarity and logical flow. We have adopted more consistent terminology when referring to the workforce, clarified that "the concept" refers to workforce composition, and provided a clearer*

*explanation of how workforce changes, expanded roles, task shifting, team composition, and organizational support relate to competence utilization. We have also strengthened the progression towards the review aim by more explicitly linking workforce composition to the organization of roles, responsibilities, and the use of available competencies in home care services. Furthermore, we have revised the statement regarding nurses as the largest regulated professional group and provided a more precise description supported by the literature. Page 4-5, lines 117, 120, 128-132, 134-140, 145-146.*

8. Paragraph 5
   The statement that organisational contexts with greater care complexity require broader and more specialised competencies would benefit from examples. What specific competencies are being referred to?
   Similarly, what is meant by "organisational structures can limit professional discretion" and "external control mechanisms restrict adaptive capacity"? These concepts are not clear to readers and would benefit from further explanation.

   *Answer: Thank you for this helpful comment. We agree that the original text required further clarification. The paragraph has been revised to provide examples of the competencies required in complex home care settings and to further explain what is meant by professional discretion, organizational structures, external control mechanisms, and adaptive capacity in the context of home care services. The relevant section has been updated accordingly. Page 5, lines 148-151, 159-166*

9. Paragraph 6
   Please check that the reference style is consistent with journal requirements. I would also have expected to see a more detailed description of the relevant 2024 review in this section rather than in the Methods.

   *Answer: Thank you for this helpful comment. We appreciate the reviewer bringing these issues to our attention. The reference formatting has been carefully reviewed and revised to ensure consistency with the journal's requirements. We also agree that a description of the recent review by Ranta and Kaunonen was more appropriately placed in the Introduction. We have therefore revised this section to provide a clearer overview of existing evidence and to better position the rationale and contribution of the present scoping review. The manuscript has been updated accordingly. Page 5-6, lines 170-173, 176-179*

10. Methods and Analysis
    Step 1

The review question could be refined. The phrase: "examining how these elements shape the distribution of tasks and responsibilities, and competence utilization" reads more like an objective than part of the review question. Would it be better to ask: “What does the international literature suggest about the organisation and composition of the home care workforce, and how do these factors influence the distribution of tasks, responsibilities, and competence utilisation?”

*Answer: Thank you for this thoughtful suggestion. We appreciate the reviewer’s proposed wording and agree that it provides a useful perspective on the formulation of the review question. We have carefully considered this suggestion and will take it into account in our ongoing refinement of the review framework and reporting. We are grateful for the reviewer’s input, which has helped us reflect on the clarity and focus of the research question.*

11. Step 2
I remain unconvinced by the rationale provided for the timeframe restriction. The justification appears to reflect a particular national context, which I was unable to identify in the cited reference. It may be sufficient to state that studies from 2015 onwards were included to capture contemporary literature on workforce competency, quality, and efficiency, which appears more consistent with the cited WHO publication.
I would also encourage the authors to make the rationale for the review easier for readers to follow. For example, rather than stating: "While Ranta and Kaunonen [9] focus specifically…"consider briefly describing the review and its key focus before explaining how the current review differs and addresses an important gap. I would also move this to the end of your introduction section.

*Answer: Thank you for this helpful comment. We have revised the rationale for the timeframe restriction to more clearly emphasize the inclusion of contemporary literature reflecting current developments in home care services. In addition, the discussion of the review by Ranta and Kaunonen has been moved to the Introduction and expanded to better clarify the contribution of the present review. The manuscript has been updated accordingly. Page 7, lines 209-213*

12. Step 6
I suggest identifying the relevant stakeholders earlier in the section. It would also be helpful to specify the country context in which the review findings are intended to be applied and how stakeholders are expected to use the findings. The rationale for including international qualitative studies could also be

developed. While I understand that qualitative studies can provide perspectives, experiences, and priorities from stakeholders, it is not clear why an international perspective is important for this review. For example, are the authors seeking to identify transferable lessons from other countries, compare workforce approaches across health and care systems, or understand the role of contextual factors in workforce organisation and care delivery? Clarifying the value of the international scope would strengthen the rationale for the review.

*Answer: Thank you for this helpful comment. We have revised the section to identify the relevant stakeholders earlier and to clarify how the review findings will inform a subsequent comparative qualitative study involving stakeholders from different countries. We have also strengthened the rationale for including international studies by emphasizing their value for understanding workforce composition and competence utilization across different health and care systems, and for identifying both transferable lessons and context-specific factors. The manuscript has been updated accordingly. Page 9, lines 256-257, 259, 262-268*

*13.* Thank you for the opportunity to review this revised manuscript. Overall, while this revision has improve the quality of the work, I would specifically suggest the authors focus on the quality of their writing and making reading of their manuscript easy for interested audiences.

*Answer: Thank you for this helpful feedback. We have carefully reviewed the manuscript and made further revisions to improve clarity, readability, and overall flow. We appreciate your suggestions and believe these changes have strengthened the quality and accessibility of the manuscript.*

---
