# Supplementary material for: Optimising workforce competence in home care services: a scoping review protocol
Source: BMJ Open. 2026 Aug 27;16(8):e118351. doi: 10.1136/bmjopen-2026-118351 (PMC13536040; doi:10.1136/bmjopen-2026-118351)
Supplement: Supplementary file 2 [file bmjopen-16-8-s001.docx]

CINAHL

((((MH (“Home Health Care+” or “Home Health Nursing”))) OR ((("Home Care Service#" or "Home Health Nursing" or "Home Health Care" or "Home Care" or "Home healthcare" or "Home based care" or "Home based nursing" or "Home based healthcare" or "Home based health care" or "Community nursing" or "Community based nursing" or "Domiciliary care" or "Home nursing"))))) AND ((((MH( "Health Labor Supply+" or "Workforce" or "Clinical Competence+" or "Scope of Practice+" or "Scope of Nursing Practice" or "Organizational Efficiency+" or "Health Resource Allocation" or "Personnel Staffing and Scheduling+" or "Task Shifting" or "Professional Role+" or "Delegation of Authority" or "Team Nursing" or "Multidisciplinary Care Team+" or "Skill Mix+"))) OR ((("Health Workforce" or "Clinical Competence" or "Scope of Practice" or "Resource Allocation" or "Task Shifting" or "Professional Role" or "Patient Care Team#" or "Health Labor Supply" or "Workforce" or "Scope of Nursing Practice" or "Organizational Efficiency" or "Health Resource Allocation" or "Personnel Staffing and Scheduling" or "Delegation of Authority" or "Team Nursing" or "Multidisciplinary Care Team#" or "Skill Mix" or "Personnel management" or "Professional standard#" or "Professional delegation" or "Nursing team#" or "Healthcare team#" or "Health care team#" or "Role optimization" or "Task allocation" or "Role distribution" or "Role responsibilities" or "Delegation" or "Care coordination" or "Job responsibilities" or "Task assignment" or "Role definition" or "Role clarity" or "Division of labor" or "Job roles" or "Job scope" or "Task distribution" or "Task sharing"))))) AND ((((("Health Personnel" or "Nurse Practitioner#" or "Nursing Staff" or "Allied Health Personnel" or "Staff Nurse#" or "Nurse#" or "Unlicensed Health Personnel" or "Home Health Aide#" or "Community Health Nurse#" or "Public Health Nurse#" or "Registered Nurse#" or "Community Health Nursing" or "Health care personnel" or "Paramedical personnel" or "Healthcare provider#" or "Health care provider#" or "Allied health professional" or "Health care support worker#" or "Healthcare support worker#" or "Assistive personnel" or "Healthcare assistant#" or "Health care assistant#" or "Licensed practical nurse#" or "LPN" or "RN" or "Medical assistant#" or "Clinical officer#" or "Community health worker#" or "Care coordinator#" or "Rehabilitation specialist#" or "Home health personnel"))) OR ((MH "Health Personnel+" OR MH "Nurse Practitioners+" OR MH "Staff Nurses" OR MH "Allied Health Personnel+" OR MH "Nurses+" OR MH "Health Personnel, Unlicensed" OR MH "Home Health Aides" OR MH "Nurses, Community Health" OR MH "Nurses, Public Health" OR MH "Registered Nurses" OR MH "Community Health Nursing"))))
